# An Evaluation of Core Decompression and Cancellous Bone Grafting for Early-Stage Avascular Necrosis of the Femoral Head

**DOI:** 10.7759/cureus.37878

**Published:** 2023-04-20

**Authors:** Harpreet Singh, Kamal Kumar Agarwal, Sangam Tyagi, Ammar Rampurwala, Amritesh Singh, Purvesh Bhrambhatt, Rishit Unjia, Neel Agarwal, Aliasgar J Rampurwala

**Affiliations:** 1 Department of Orthopaedics, Geetanjali Medical College & Hospital, Udaipur, IND; 2 Department of Orthopaedics, University College of Medical Sciences, Guru Teg Bahadur Hospital, Delhi, IND; 3 Department of Orthopaedics, Parul Institute of Medical Sciences and Research, Vadodara, IND

**Keywords:** harris hip score, vas score, iliac crest, core decompression, avascular necrosis of femoral head

## Abstract

Background

Avascular necrosis (AVN) is characterized by bone death due to impaired blood supply leading to its collapse causing pain and suboptimal joint function. The blood supply of the femoral head is so tenuous that even a slight vascular injury can predispose to AVN. Hence, AVN is commonly seen in the femoral head. Core decompression can arrest or even reverse the process of AVN and can avoid femoral head collapse and its sequelae. A lateral trochanteric approach is used for core decompression. The necrotic bone is removed from the femoral head. The use of a non-vascularized bone graft is more attractive than a vascularized graft because it is significantly less technically challenging. The regenerative properties due to the presence of osteoblasts in the trabecular bone and the ability to procure a large amount of graft make the iliac crest the gold standard site of cancellous bone graft harvesting. Core decompression can be considered an effective treatment modality in early-stage AVN (up to stage 2B) of the femoral head.

Methodology

A prospective, interventional study was conducted in a tertiary care teaching hospital in southern Rajasthan, India. 20 Patients with AVN of the femoral head (up to grade 2B of Ficat and Arlet classification) who met the inclusion and exclusion criteria and presented to the orthopedic outpatient department of our institute were included in this study. Patients were treated with core decompression and cancellous bone grafting with a graft taken from the iliac crest. The Harris Hip Score (HHS) and Visual Analog Scale (VAS) score were used to assess the outcomes.

Results

In our study, the majority (50%) of the patients were in the 20-30-year age group, making it the most common age group with a male predominance (85%). In this study, the final result was calculated according to the HHS and VAS scores. The mean HHS was 69.45 preoperatively and 83.55 at six months postoperatively. Similarly, the mean VAS score was 6.3 preoperatively and 3.8 at six months postoperatively.

Conclusions

Core decompression with cancellous bone grafting is a promising procedure in stages 1 and 2 as it reduces the symptoms in the majority of cases and improves functional outcomes.

## Introduction

Avascular necrosis (AVN) is characterized by bone death as a result of impaired blood supply leading to its collapse causing pain and suboptimal joint function [1]. Although the most common cause is idiopathic, the pathology can be caused by trauma, infections, tumors, corticosteroids, alcohol abuse, smoking, sickle cell disease, radiation therapy, dysbarism, and connective tissue disorders. Branches of the profunda femoral artery supply the femoral head. The blood supply of the femoral head is so tenuous that even a slight vascular injury can predispose to AVN [2]. Hence, AVN is commonly seen in the femoral head. Pain may be mild at onset, but if not intervened at an early stage, it may worsen gradually and interfere with the activities of daily living.

The increased intraosseous pressure can be relieved by core decompression, thereby providing pain relief to patients [3]. Core decompression is used both as a diagnostic and therapeutic procedure [4]. Core decompression leads to creeping substitution in the necrotic area by opening up the space for vascular channels in the affected area and decreasing the intraosseous pressure. Core decompression can arrest or even reverse the process of AVN and can avoid femoral head collapse and its sequelae. Core decompression can preserve the femoral head at an early stage of the pathology [5].

A lateral trochanteric approach is used for core decompression. The necrotic bone is removed from the femoral head [6]. A vascularized or non-vascularized bone graft can be used to fill the space thus formed.

The use of a non-vascularized bone graft is more attractive than that of a vascularized graft because it is significantly less technically challenging. Non-vascularized autologous bone grafting has several theoretical advantages. This method leads to the decompression of the avascular lesion and the elimination of the necrotic bone, breaking the cycle of ischemia and intraosseous hypertension, in addition to providing growth factors from the graft [7].

The regenerative properties due to the presence of osteoblasts in the trabecular bone and the ability to procure a large amount of graft make the iliac crest the gold standard site of cancellous bone graft harvesting [8,9].

Core decompression can be considered an effective treatment modality in the early stages (up to stage 2B) of AVN of the femoral head [10]. Therefore, this study was conceptualized to study the effect of core decompression along with cancellous bone grafting on cases of AVN of the femoral head.

## Materials and methods

A prospective, interventional study was conducted in a tertiary care teaching hospital in southern Rajasthan, India from February 2021 to June 2022 after obtaining approval from the Institutional Research Review Committee, Geetanjali Medical College & Hospital (approval number: GMCH/IRRC/PG20/2021/5603(37). A total of 20 patients with AVN of the femoral head (up to grade 2B of Ficat and Arlet classification) who met the inclusion and exclusion criteria were included in this study. The inclusion and exclusion criteria are presented in Table 1.

**Table 1:** Inclusion and exclusion criteria.

Inclusion criteria	Exclusion criteria
Patients with avascular necrosis of the femoral head up to stage 2b (Ficat and Arlet)	Previous infection of the hip joint
Patients aged between 18 and 60 years	Neuromuscular disorder of the hip joint
Patients providing consent for the surgical procedure and participation in the study	Severe osteoporosis

AVN stage was confirmed preoperatively using an X-ray of the pelvis with both hip joints and an MRI of both hips. A preoperative workup was done and written and informed consent was taken. After taking written and informed consent, patients were taken for the operation.

Surgical procedure

**Figure 1:**
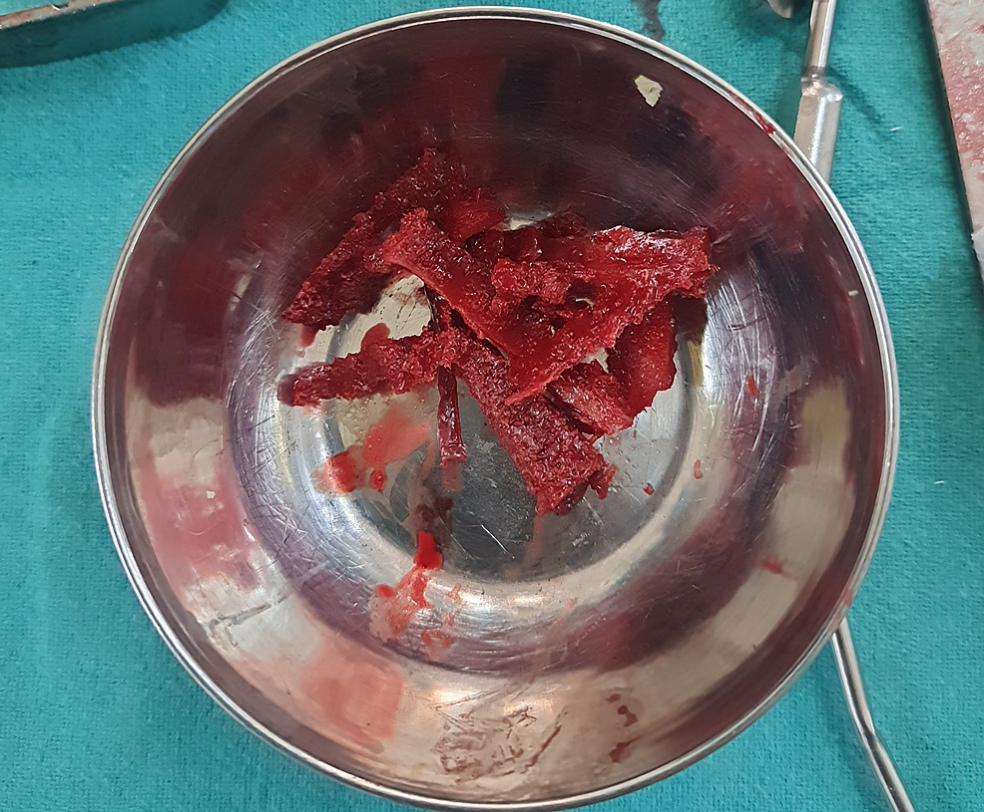
Bone graft taken from the iliac crest.

Patients were placed on a traction table in a supine position. Sterile painting and draping were done. Subsequently, a 2-3 cm mid-lateral longitudinal incision was made over the subtrochanteric region. The tensor fascia lata was split in the direction of its fibers, and the vastus lateralis muscle was elevated. Under C-arm guidance, a 3.2 mm threaded guide pin was inserted through the lateral cortex into the affected part of the femoral head, with the entry point between the lesser trochanter and greater trochanter. The guide pin was directed toward the center of the necrotic area of the femoral head. The guide pin was over-reamed with an 8 mm reamer. The cancellous bone graft was taken from the outer table of the iliac crest of the patient (Figure 1).

The gap created with the cancellous bone graft was packed. Both the operative sites were washed thoroughly with normal saline and layer-wise closure was done with vicryl and ethilon. Finally, sterile dressing was done.

Other available medical and surgical treatments

Non-weight bearing and use of skin traction, lipid-lowering agents, anticoagulants, vasodilators, and bisphosphonates can be used for treating early AVN but the results of this medical management are questionable. Other than medical treatment, a few surgical treatments are also available such as muscle pedicle bone graft, vascular bone graft, osteotomies, stem cell transplantation, and, in advanced stages (Ficat and Arlet III and IV), total hip replacement.

Postoperative management

Intravenous (IV) antibiotics were given till the third postoperative day, followed by oral antibiotics until suture removal. All patients were immobilized for 1.5 months postoperatively. Dressings were done on the first, third, and fifth postoperative days. Physiotherapy started on day one which included knee mobilization, static quadriceps exercises, ankle pump, and toe movement. After 1.5 months, partial weight bearing was started.

Follow-up interval

Follow-up was done at 1.5 months, three months, and, finally, six months. Patients were assessed using the Harris Hip Score (HHS) and Visual Analog Scale (VAS) scores at follow-up.

## Results

This study was conducted in the Department of Orthopaedics, Geetanjali Medical College & Hospital, Udaipur from February 2021 to June 2022. Based on the selection criteria, 20 patients with AVN of the femoral head (up to Ficat and Arlet stage 2b) were selected. In our study, the majority (50%) of patients were in the 20-30-year age group, making it the most common age group, followed by the 31-40-year age group (40%) (Table 2).

**Table 2:** Age, sex, and duration of pain distribution.

Characteristics	Parameters	Number of patients
Age distribution (in years)	20–30	10
	31–40	8
	41–50	1
	51–60	1
Sex distribution	Male	17
	Female	03
Duration of pain (in months)	0–3 months	11
	4–6 months	06
	7–9 months	02
	10–12 months	01

There was a male predominance, with 17 out of 20 patients being male. All patients became symptomatic within the last year (range = 2-12 months). Of 20 patients, 11 presented to us within three months of developing symptoms (Table 2). Of 20 patients, 10 were able to sit cross-legged, squat, and climb stairs without any significant problems. Four patients were not able to sit cross-legged or squat, while five patients were not able to climb stairs.

Lifestyle modification was advised to all patients. Five patients who were alcoholics were advised to stop taking alcohol. Seven patients had a history of steroid use due to COVID-19 infection. No specific etiology was identified in eight patients (Table 3).

**Table 3:** Etiology/Risk factors for avascular necrosis of the femoral head.

Risk factors/Etiology	Number of patients
Idiopathic	8
Steroid	7
Alcohol	5

HHS

The mean HHS was 69.45 preoperatively and 83.55 at six months postoperatively (p ≤ 0.0003) (Table 4).

**Table 4:** Harris Hip Score preoperative and at six months postoperatively.

Degree	Preoperative	At 6 months
≤70	9	4
70–80	7	2
81–90	4	5
>90	0	9

VAS score

The mean VAS score was 6.3 preoperatively and 3.8 at six months postoperatively (p ≤ 0.0001) (Table 5).

**Table 5:** Visual Analog Scale score preoperatively and at six months postoperatively.

VAS score	Preoperative	At 6 months postoperatively
0–3	0	12
4–6	14	4
7–9	6	4

Complications

Overall, 30% of patients developed secondary arthritis after six months and progressed to Ficat and Arlet stage 3/4. In total, 17 patients had pain at the donor site immediately after surgery. After three months, only one patient had pain at the donor site which was managed with analgesics. After six months, no patients had pain at the donor site. Apart from this, no significant donor site morbidity was seen (Table 6).

**Table 6:** Complications.

Complication	Number of patients
Secondary arthritis	6
Pain at the graft donor site (postoperatively)	17
Pain at the donor site (at 1.5 months)	5
Pain at the donor site (at 3 months)	1
Pain at the donor site (at 6 months)	0

Radiographs

**Figure 2:**
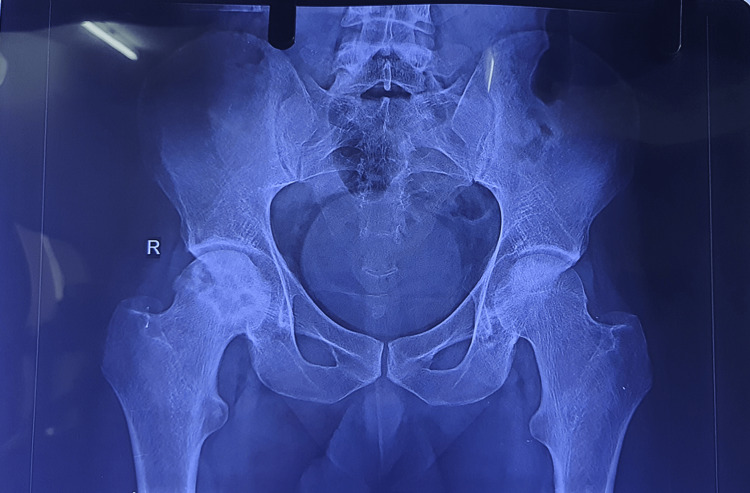
Preoperative X-ray of the patient showing avascular necrosis of the femoral head.

**Figure 3:**
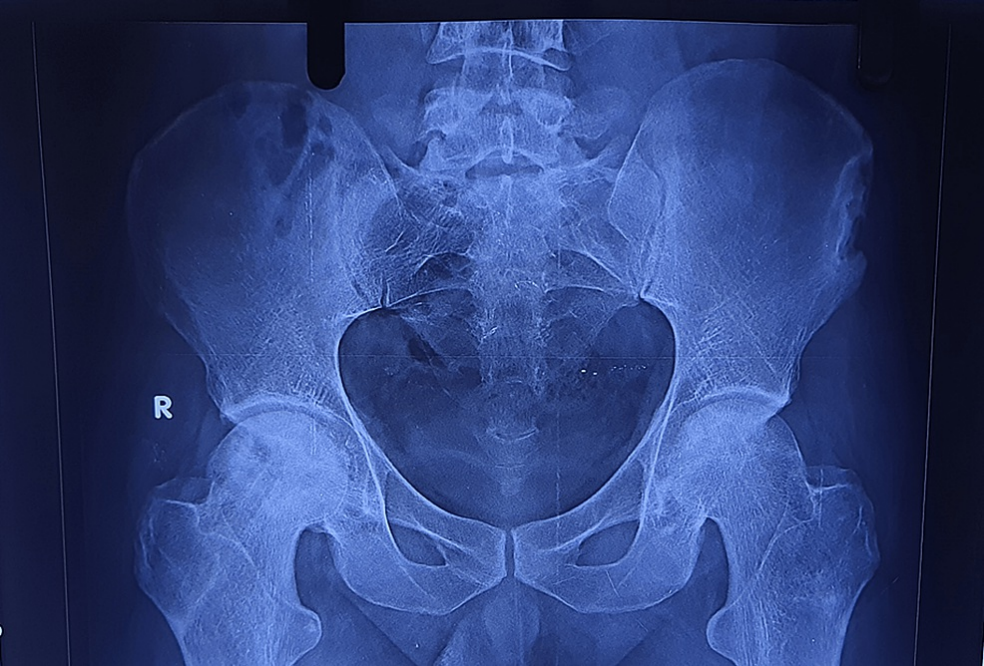
Immediate postoperative X-ray.

**Figure 4:**
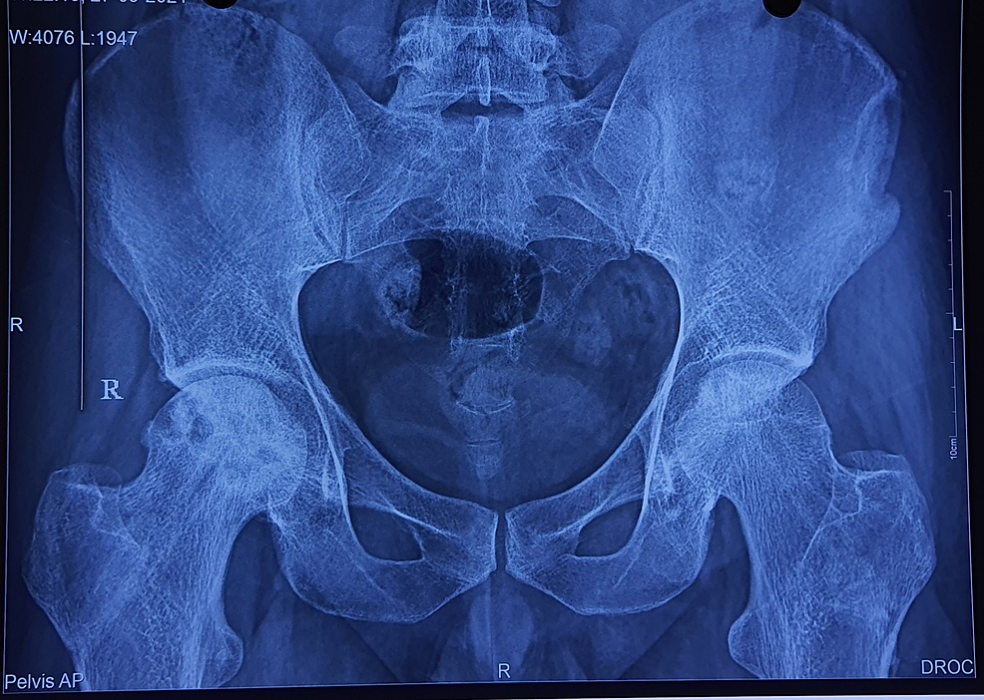
X-ray at 1.5 months of follow-up.

**Figure 5:**
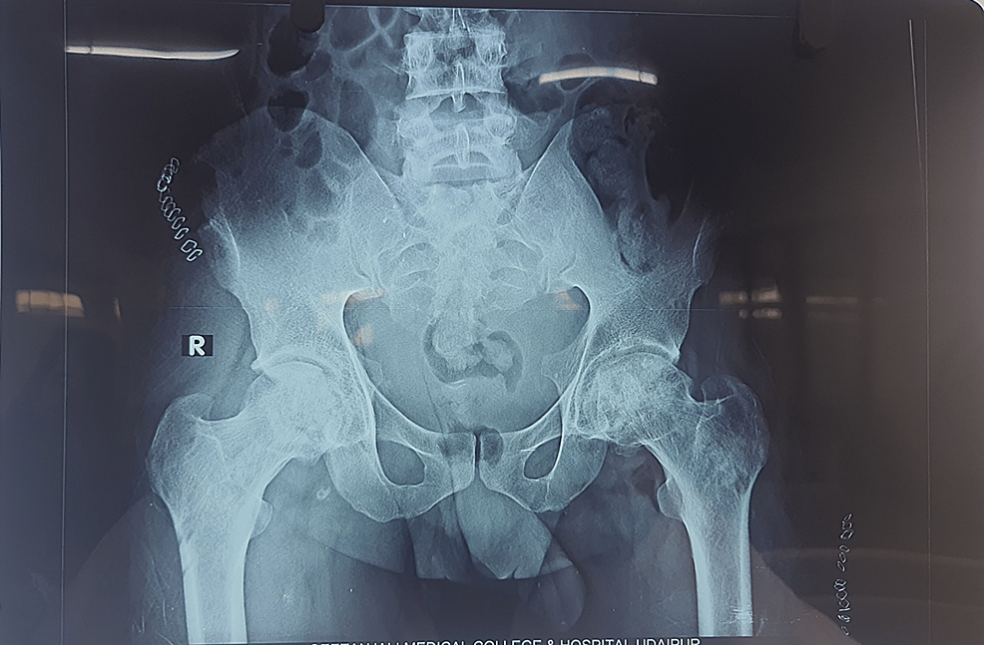
X-ray at three months of follow-up.

**Figure 6:**
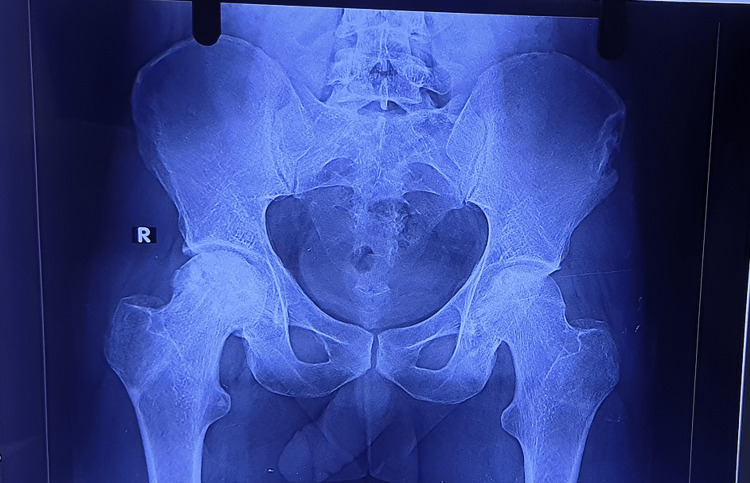
X-ray at the final follow-up.

Figures 2-6 are the radiographic images of one of the study patients showing preoperative, postoperative, and follow-up X-rays. On radiographic evaluation, the patient operated on for AVN of the femoral head with core decompression and cancellous bone grafting showed significant improvement on the six-month follow-up.

## Discussion

The blood supply of the femoral head is so tenuous that even a slight vascular injury can predispose to AVN. Hence, AVN is commonly seen in the femoral head. Studies have shown that core decompression can arrest or even reverse the process of AVN and can avoid femoral head collapse and its sequelae. Core decompression can preserve the femoral head at an early stage of the pathology [4,5].

Steinberg et al. reported that 92% of patients who underwent non-operative management had femoral head collapse at the end of two years. They found that patients with early-stage AVN who had undergone core decompression and cancellous bone graft had about a 70% success rate [11].

Core biopsies were performed in the early 60s in a small number of patients with AVN of the femoral head to examine the pathological changes. The increased intraosseous pressure was relieved by core decompression, thereby providing pain relief to the patient.

According to Steinberg, treating early AVN of the femoral head with core decompression and cancellous bone grafting is a safe and efficient method [12].

We studied 20 hips having AVN of the femoral head treated by core decompression and autologous cancellous bone grafting with a graft taken from the iliac crest.

In a study, Babhulkar [13] treated 32 patients with AVN of the femoral head by core decompression and iliac crest grafting and reported pain relief in all patients. Similar results were found in our studies as well. At the final follow-up, 16 patients had pain relief.

Core decompression with vascularized fibular graft has been the treatment of choice in cases of early AVN. Vascularized fibular grafting not only provides subchondral support but also vascularization at the site. However, the disadvantage is morbidity at the donor site and the need for microvascular anastomosis [14]. Therefore, a cancellous graft from the iliac crest has been considered as an alternative. Osteogenesis and osteoinduction are two of the key characteristics of bone grafts, and they are frequently attributed to cancellous grafts rather than cortical grafts. This makes it advantageous for revascularization as well. Moreover, it avoids donor site morbidity associated with fibular graft. A densely filled graft may provide structural stability [15,16]. Therefore, we preferred core decompression with autologous cancellous bone grafting with a graft taken from the iliac crest.

In our study, all patients were kept non-weight bearing for 1.5 months, and after that partial weight bearing was started. This was done because the femoral head would need enough time for regeneration after core decompression. Therefore, if weight bearing is started before sufficient bone growth, it could result in a collapse of the femoral head due to stress [13].

In our study, 90% of the patients were in the 20-40-year age group, and the remaining 10% of patients were in the 41-60-year age group. Our study showed a male predominance as 17 (85%) patients were male. Similar male predominance was also reported by Babhulkar [13] where 81.25% of patients were males.

Activities of daily living were assessed by cross-legged sitting, squatting, and stair climbing. Overall, 50% of patients faced difficulty in these activities, suggesting that activities of daily living are affected even in the early stages of AVN.

he most common stage according to Ficat and Arlet classification at which the patients presented was stage 2B (45%), followed by stage 2A (40%) and stage 1 (15%). No difference was noted in the preoperative and postoperative MRI stages in 11 patients over six months. Three patients reverted from stage 2B to 2A on MRI grading. Six patients showed progression to later stages on MRI grading.

The mean HHS was 69.45 preoperatively and 83.55 at six months postoperatively. Out of 20 hips, 14 had excellent (HHS >90) to good (HHS 80-90) outcomes, while six had fair (HHS 70-80) to poor (HHS >70) outcomes.

The mean VAS score was 6.3 preoperatively and 3.8 at six months postoperatively. Out of 20 patients, 16 had excellent to good outcomes and four had fair to poor outcomes in terms of pain relief.

Although no significant improvement was seen in the MRI findings, as interpreted by HHS results, excellent functional outcomes were found in 66% of grade 1 patients, 50% of grade 2A patients, and 33% of grade 2B patients. Poor outcomes were found in 33% of grade 1 patients, 12.5% of grade 2A patients, and 22% of grade 2B patients.

Therefore, our study suggests that core decompression with autologous cancellous bone grafting might not reverse AVN progression but can delay the progression of AVN, avoid collapse, reduce pain, and provide improvement in functional outcomes at least in the short term.

Limitations

The study had a relatively shorter follow-up period of six months. Probably, a longer follow-up is needed for evaluating long-term functional outcomes. A shorter follow-up period was because of time constraints. Another limitation was the small sample size as overestimation of treatment effect is more likely in a small sample compared to a large sample. Patient compliance was also a limitation of our study. The limitation of the surgical method was that it could only be done in the early stage of the disease (Ficat and Arlet stages I and II) and did not have any significant outcomes if done in the advanced stages of the disease (Ficat and Arlet stages III and IV). Other surgical treatments such as muscle pedicle bone grafting and vascular grafting were not done due to time, technical, and financial constraints.

## Conclusions

Core decompression with cancellous bone grafting is a promising procedure in stages 1 and 2 as it reduces the symptoms in the majority of cases and provides improvement in functional outcomes.

## References

[1] Mont MA, Jones LC, Hungerford DS (2006). Nontraumatic osteonecrosis of the femoral head: ten years later. J Bone Joint Surg Am.

[2] Sharma D, Maulik Jhaveri D, Urang Patel D, Jha A, Shah P, Golwala P (2019). Pain relief with core decompression and autologous bone graft in osteonecrosis of femoral head in grade 2. Int J Orthop Sci.

[3] Hua KC, Yang XG, Feng JT, Wang F, Yang L, Zhang H, Hu YC (2019). The efficacy and safety of core decompression for the treatment of femoral head necrosis: a systematic review and meta-analysis. J Orthop Surg Res.

[4] Lavernia CJ, Sierra RJ (2000). Core decompression in atraumatic osteonecrosis of the hip. J Arthroplasty.

[5] Urbaniak JR, Coogan PG, Gunneson EB, Nunley JA (1995). Treatment of osteonecrosis of the femoral head with free vascularized fibular grafting. A long-term follow-up study of one hundred and three hips. J Bone Joint Surg Am.

[6] Tan Y, He H, Wan Z (2020). Study on the outcome of patients with aseptic femoral head necrosis treated with percutaneous multiple small-diameter drilling core decompression: a retrospective cohort study based on magnetic resonance imaging and equivalent sphere model analysis. J Orthop Surg Res.

[7] Banal J, Sohal H, Sharma R, Dhake J, Singh G (2017). Autologous (Non vascularized) fibular grafting with cancellous bone grafting for treatment of femoral head osteonecrosis (Ficat and Arlet stage 1 2a 2b 3). Int J Orthop Sci.

[8] Park JJ, Hershman SH, Kim YH (2013). Updates in the use of bone grafts in the lumbar spine. Bull Hosp Jt Dis (2013).

[9] Khan SN, Cammisa FP Jr, Sandhu HS, Diwan AD, Girardi FP, Lane JM (2005). The biology of bone grafting. J Am Acad Orthop Surg.

[10] Schwarz Lausten G (1999). Non-traumatic necrosis of the femoral head. Int Orthop.

[11] Steinberg ME, Larcom PG, Strafford B, Hosick WB, Corces A, Bands RE, Hartman KE (2001). Core decompression with bone grafting for osteonecrosis of the femoral head. Clin Orthop Relat Res.

[12] Steinberg ME (1995). Core decompression of the femoral head for avascular necrosis: indications and results. Can J Surg.

[13] Babhulkar S (2009). Osteonecrosis of femoral head: treatment by core decompression and vascular pedicle grafting. Indian J Orthop.

[14] Yoo MC, Kim KI, Hahn CS, Parvizi J (2008). Long-term followup of vascularized fibular grafting for femoral head necrosis. Clin Orthop Relat Res.

[15] Sen RK (2009). Management of avascular necrosis of femoral head at pre-collapse stage. Indian J Orthop.

[16] Sanap A, Rabari YB, Teja V, Shah S (2018). Management of avascular necrosis of femoral head by core decompression. Int J Res Orthop.

